# Toxicological knowledge discovery by mining emerging patterns from toxicity data

**DOI:** 10.1186/1758-2946-5-S1-O9

**Published:** 2013-03-22

**Authors:** Richard Sherhod, Valerie J Gillet, Thierry Hanser, Philip N Judson, Jonathan D Vessey

**Affiliations:** 1 Information School, The University of Sheffield, Sheffield, S1 4DP, UK; 2 Lhasa Limited, Leeds, LS2 9HD, UK

## 

Predicting the risk of toxic and environmental effects of chemical compounds is of great importance to all chemical industries [1]. Expert systems have shown success in predicting toxic risk by applying established knowledge of toxicology encoded as a knowledge base of structural alerts and a reasoning model. A disadvantage of expert systems is that developing new structural alerts requires considerable time and effort from domain experts. In order to expedite this process a software tool has been developed that can automatically mine representations of activating features directly from toxicity datasets and present them in an interpretable form.

Our knowledge discovery tool applies emerging pattern (EP) mining [2]: a form of association rule mining [3] that is well known to computer science, but is relatively new to chemistry [4]. The EP mining algorithm accepts any data expressed as a series of binary properties, which is divided into two classes, and extracts patterns of those properties that are frequent within the data and are more frequent in one data class compared to the other. By mining emerging patterns from toxicity datasets, encoded as fingerprints of binary descriptors, the tool generates patterns of features that distinguish toxicants from innocuous compounds. These patterns represent potentially activating features of the toxic compounds that may then be used to define new alerts.

The knowledge discovery tool has been tested using a public dataset of 3489 mutagens and 2981 non-mutagens, encoded as fingerprints of approximately 2000 functional groups and ring descriptors. EPs were produced and grouped into a number of hierarchical families. Six of the EPs that represented distinct chemical classes were selected for manual inspection by a toxicology expert. Relevant literature was analysed to find a mechanistic rationale for the mined features, which resulted in four new structural alerts for *in vitro *mutagenicity.

## References

[B1] Cronin MTD Madden JC In Silico Toxicology: Principles and Applications Royal Society of Chemistry 2010

[B2] Dong G Li J Efficient Mining of Emerging Patterns: Discovering Trends and Differences Proceedings of the Fifth ACM SIGKDD International Conference on Knowledge Discovery and Data Mining: 15-18 August 1999 1999 San Diego. ACM Press 43 52

[B3] Agrawal R Imieliŕski T Swami A Mining Association Rules between Sets of Items in Large Databases Proceedings of the 1993 ACM SIGMOD International Conference on Management of Data 1993 Washington DC. AMC Press 207 216 26-28 May 1993

[B4] Auer J Bajorath J Emerging Chemical Patterns: A New Methodology for Molecular Classification and Compound Selection J Chem Inf Mod 2006 46 2502 2514 10.1021/ci600301t 17125191

